# Reprogramming of RNA m^6^A Modification Is Required for Acute Myeloid Leukemia Development

**DOI:** 10.1093/gpbjnl/qzae049

**Published:** 2024-06-24

**Authors:** Weidong Liu, Yuhua Wang, Shuxin Yao, Guoqiang Han, Jin Hu, Rong Yin, Fuling Zhou, Ying Cheng, Haojian Zhang

**Affiliations:** State Key Laboratory of Oral & Maxillofacial Reconstruction and Regeneration, Key Laboratory of Oral Biomedicine Ministry of Education, Hubei Key Laboratory of Stomatology, School & Hospital of Stomatology, Wuhan University, Wuhan 430079, China; State Key Laboratory of Oral & Maxillofacial Reconstruction and Regeneration, Key Laboratory of Oral Biomedicine Ministry of Education, Hubei Key Laboratory of Stomatology, School & Hospital of Stomatology, Wuhan University, Wuhan 430079, China; Frontier Science Center for Immunology and Metabolism, Medical Research Institute, Wuhan University, Wuhan 430071, China; Frontier Science Center for Immunology and Metabolism, Medical Research Institute, Wuhan University, Wuhan 430071, China; Department of Hematology, Zhongnan Hospital, Medical Research Institute, Wuhan University, Wuhan 430071, China; Frontier Science Center for Immunology and Metabolism, Medical Research Institute, Wuhan University, Wuhan 430071, China; Frontier Science Center for Immunology and Metabolism, Medical Research Institute, Wuhan University, Wuhan 430071, China; Department of Hematology, Zhongnan Hospital, Medical Research Institute, Wuhan University, Wuhan 430071, China; Department of Hematology, Zhongnan Hospital, Medical Research Institute, Wuhan University, Wuhan 430071, China; Frontier Science Center for Immunology and Metabolism, Medical Research Institute, Wuhan University, Wuhan 430071, China; School of Life Sciences, Zhengzhou University, Zhengzhou 450001, China; State Key Laboratory of Oral & Maxillofacial Reconstruction and Regeneration, Key Laboratory of Oral Biomedicine Ministry of Education, Hubei Key Laboratory of Stomatology, School & Hospital of Stomatology, Wuhan University, Wuhan 430079, China; Frontier Science Center for Immunology and Metabolism, Medical Research Institute, Wuhan University, Wuhan 430071, China; Department of Hematology, Zhongnan Hospital, Medical Research Institute, Wuhan University, Wuhan 430071, China; Taikang Center for Life and Medical Sciences, Wuhan University, Wuhan 430071, China

**Keywords:** RNA m^6^A modification, Hematopoiesis, Acute myeloid leukemia, Leukemia-initiating cell, ATP-binding cassette subfamily D member 2

## Abstract

Hematopoietic homeostasis is maintained by hematopoietic stem cells (HSCs), and it is tightly controlled at multiple levels to sustain the self-renewal capacity and differentiation potential of HSCs. Dysregulation of self-renewal and differentiation of HSCs leads to the development of hematologic diseases, including acute myeloid leukemia (AML). Thus, understanding the underlying mechanisms of HSC maintenance and the development of hematologic malignancies is one of the fundamental scientific endeavors in stem cell biology. *N*^6^-methyladenosine (m^6^A) is a common modification in mammalian messenger RNAs (mRNAs) and plays important roles in various biological processes. In this study, we performed a comparative analysis of the dynamics of the RNA m^6^A methylome of hematopoietic stem and progenitor cells (HSPCs) and leukemia-initiating cells (LICs) in AML. We found that RNA m^6^A modification regulates the transition of long-term HSCs into short-term HSCs and determines the lineage commitment of HSCs. Interestingly, m^6^A modification leads to reprogramming that promotes cellular transformation during AML development, and LIC-specific m^6^A targets are recognized by different m^6^A readers. Moreover, the very long chain fatty acid transporter ATP-binding cassette subfamily D member 2 (ABCD2) is a key factor that promotes AML development, and deletion of *ABCD2* damages clonogenic ability, inhibits proliferation, and promotes apoptosis of human leukemia cells. This study provides a comprehensive understanding of the role of m^6^A in regulating cell state transition in normal hematopoiesis and leukemogenesis, and identifies ABCD2 as a key factor in AML development.

## Graphical abstract

**Graphical abstract:**
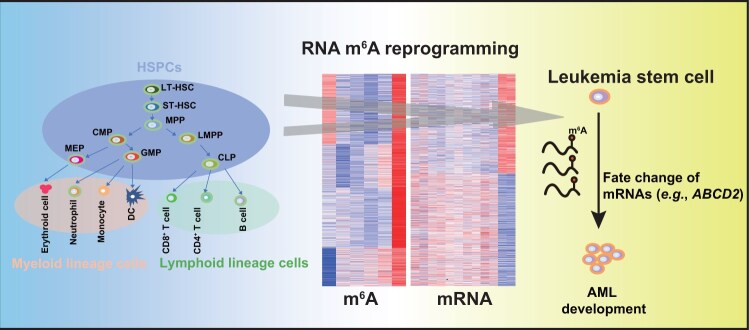

## Introduction

In the blood system, homeostasis is maintained by hematopoietic stem cells (HSCs), which routinely replenish all blood cell lineages throughout the lifetime of an individual, and this process is tightly controlled at different levels to sustain the self-renewal capacity and differentiation potential of HSCs [1–3]. Increasing evidence reveals the heterogeneity of HSCs at the transcriptional level [4]. Thus, understanding how the transcriptional states of HSCs are regulated is one of the fundamental scientific endeavors in stem cell biology.

Generally, genetic or epigenetic alterations in hematopoietic cells, especially hematopoietic stem and progenitor cells (HSPCs), may interfere with the regulation of hematopoietic homeostasis, which could lead to the development of various hematologic diseases, such as leukemia and myelodysplastic syndrome. Among the hematologic diseases, acute myeloid leukemia (AML) is a fatal hematologic malignancy characterized by uncontrolled expansion and arrested differentiation of myeloid progenitors [5,6]. The development of AML is associated with the accumulation of acquired genetic alterations in HSPCs [7–9], resulting in the transformation of HSPCs into leukemia-initiating cells (LICs). LICs are responsible for leukemia initiation, progression, and relapse [10–13]. Currently, the 5-year overall survival of adult AML patients is approximately 30%, and successful therapeutic treatment of AML patients remains a big challenge. Therefore, it is necessary to further investigate the molecular mechanisms of LIC maintenance and to gain a deeper understanding of AML development, which might bring hope for the development of effective therapeutic strategies.

*N* ^6^-methyladenosine (m^6^A) modification is the most common messenger RNA (mRNA) modification, and it plays important roles in mRNA fate determination by regulating RNA splicing, transport, localization, stability, translation, and degradation [14–18]. RNA m^6^A modification is dynamically reversible; it is installed by a methyltransferase complex (also called writer) and removed by the m^6^A demethylase ALKBH5 or FTO (also called eraser). The methyltransferase complex consists of two core subunits, METTL3 and METTL14, along with several regulatory subunits, including WTAP, RBM15/15B, VIRMA, CBLL1, and ZC3H13 [19–24]. Both ALKBH5 and FTO belong to the ALKB family, and their function depends on Fe(II) and alpha-ketoglutarate [25,26]. Normally, m^6^A sites are recognized by different m^6^A readers, including YTH domain-containing protein 1/2 (YTHDC1/2) [27,28], YTH domain-containing family members 1–3 (YTHDF1/2/3) [29,30], and insulin-like growth factor-2 mRNA-binding protein (IGF2BP) family members 1–3 (IGF2BP1/2/3) [31]. These m^6^A readers and their associated protein machineries are essential for the biological function of RNA m^6^A modification under various physiological and pathological contexts [32].

Increasing evidence demonstrates that RNA m^6^A modification plays a key role in regulating normal hematopoiesis and hematologic malignancies [33–42]. Recently, we deciphered RNA m^6^A methylome in hematopoiesis and leukemogenesis and uncovered the essential role of m^6^A in determining the transcriptional state of HSCs and LICs [4,43]. For instance, from the landscape of RNA m^6^A modification across the hematopoietic system, we found that m^6^A modification is cell-type-specific and demonstrates dynamic patterns across the hematopoietic hierarchy. Interestingly, m^6^A modification is necessary for stabilizing a great subset of mRNAs that are key in maintaining the function of HSCs. Moreover, we found that RNA m^6^A is greatly altered during leukemogenesis and that approximately 60% of m^6^A targets are found only in LICs, indicating an obvious change in cellular state during leukemogenesis. These findings suggest that m^6^A plays unexpected and much more complex roles in hematopoiesis and leukemogenesis. Thus, it is necessary to further perform m^6^A profiling and explore the role of m^6^A in hematopoiesis and leukemogenesis. We explored the dynamics of RNA m^6^A methylome in normal HSPCs and LICs and determined the key role of m^6^A modification in the regulation of the cellular state of HSCs and LICs. This work provides a new and detailed insight into hematopoiesis and leukemogenesis.

## Results

### RNA m^6^A modification regulates the transition of long-term HSCs into short-term HSCs

**Figure 1:**
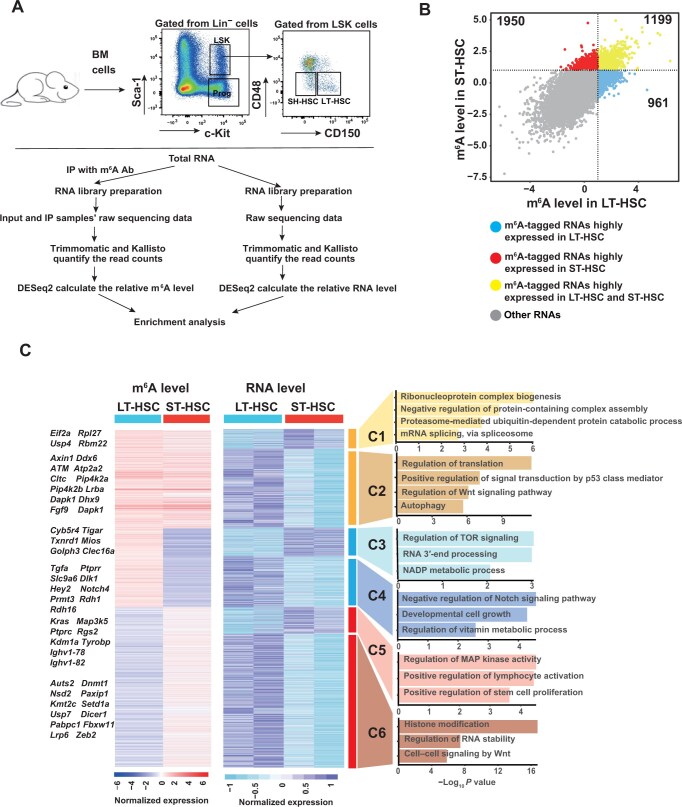
RNA m^6^A modification regulates the transition from LT-HSCs to ST-HSCs **A**. Schematic of SLIM-seq and gating strategy of HSCs. **B**. Scatter plot showing the m^6^A-tagged RNAs highly expressed in both LT-HSCs and ST-HSCs. The global correlation for m^6^A levels between LT-HSCs and ST-HSCs is 0.6909. **C**. Comprehensive correlation and GO enrichment analysis of m^6^A and RNA levels in LT-HSCs and ST-HSCs. Left: heatmap showing the m^6^A and RNA levels of 4110 m^6^A-tagged genes. Right: GO enrichment analysis results for Clusters 1–6. SLIM-seq, super-low-input m^6^A sequencing; HSC, hematopoietic stem cell; LT-HSC, long-term HSC; ST-HSC, short-term HSC; GO, Gene Ontology; IP, immunoprecipitation; Lin^−^, lineage-negative; LSK, Lin^−^Sca-1^+^c-Kit^+^; Ab, antibody; BM, bone marrow.

We recently developed a highly sensitive and efficient super-low-input m^6^A sequencing (SLIM-seq) strategy for rare HSCs and established a comprehensive m^6^A landscape across the hematopoietic hierarchy [4]. Interestingly, compared with long-term HSCs (LT-HSCs), short-term HSCs (ST-HSCs) contained up to 62% new m^6^A modifications, implying a sharp transition in cellular state as LT-HSCs differentiate into ST-HSCs. This observation drove us to further investigate this transition in LT-HSCs and ST-HSCs at the m^6^A modification level. We further analyzed our SLIM-seq data by focusing on the 2160 and 3149 m^6^A-tagged mRNAs identified in LT-HSCs and ST-HSCs, respectively (**Figure 1**A). Among these m^6^A targets, 1199 were found both in LT-HSCs and ST-HSCs, 961 were LT-HSC-specific, and 1950 were ST-HSC-specific (Figure 1B).

To investigate the role of m^6^A modification in determining mRNA fates in HSCs, we analyzed the m^6^A landscape in relation to mRNA expression level in LT-HSCs and ST-HSCs (Figure 1C). Considering the correlation between m^6^A and mRNA levels in LT-HSCs and ST-HSCs, we grouped the m^6^A mRNAs into six clusters (Figure 1C). Clusters 1 and 2 comprised the overlapping m^6^A-tagged mRNAs showing high relative mRNA expression levels in ST-HSCs and LT-HSCs, respectively. Clusters 3 and 4 consisted of the LT-HSC-enriched m^6^A targets with high relative mRNA expression levels in ST-HSCs and LT-HSCs, respectively. Clusters 5 and 6 consisted of the ST-HSC-enriched m^6^A mRNAs with high relative mRNA expression levels in ST-HSCs and LT-HSCs, respectively. We took a closer look into Clusters 3–6 given their different m^6^A modifications in LT-HSCs and ST-HSCs. Gene Ontology (GO) analysis showed the enrichment of several signaling pathways and biological processes in these clusters (Figure 1C). For instance, genes in clusters 3 and 5 were highly expressed in ST-HSCs, and they are mainly involved in the regulation of mechanistic target of rapamycin (mTOR) signaling, RNA 3′-end processing, nicotinamide adenine dinucleotide phosphate (NADP) metabolic process, regulation of mitogen-activated protein kinase (MAPK) activity, positive regulation of lymphocyte activation, and positive regulation of stem cell proliferation. These pathways are closely related to the biological behaviors of ST-HSCs. Intriguingly, compared with those in LT-HSCs, the genes enriched during lymphocyte activation (*e.g.*, *Ikzf1*, *Runx3*, *Dpp4*, *Tyrobp*, and *Tnfrsf4*) showed higher mRNA expression but lower m^6^A levels in ST-HSCs, suggesting that m^6^A modification is involved in lymphoid differentiation of HSCs (Figure 1C). The genes in Clusters 4 and 6 showed high expression levels in LT-HSCs, and they are mainly enriched in vitamin metabolic process, regulation of Notch signaling, developmental cell growth, histone modification, regulation of RNA stability, and Wnt signaling. In addition, key epigenetic regulatory genes (*e.g.*, *Dnmt1*, *Nsd2*, *Kmt2c*, and *Setd1a*) and Wnt signaling genes (*e.g.*, *Fbxw11*, *Lrp6*, and *Zeb2*) showed higher mRNA expression levels but lower m^6^A levels in LT-HSCs than in ST-HSCs (Figure 1C). Collectively, these findings indicate that m^6^A modification plays a role in the control of the transcriptional state of HSCs.

### RNA m^6^A modification is involved in myeloid and lymphoid lineage commitment

**Figure 2:**
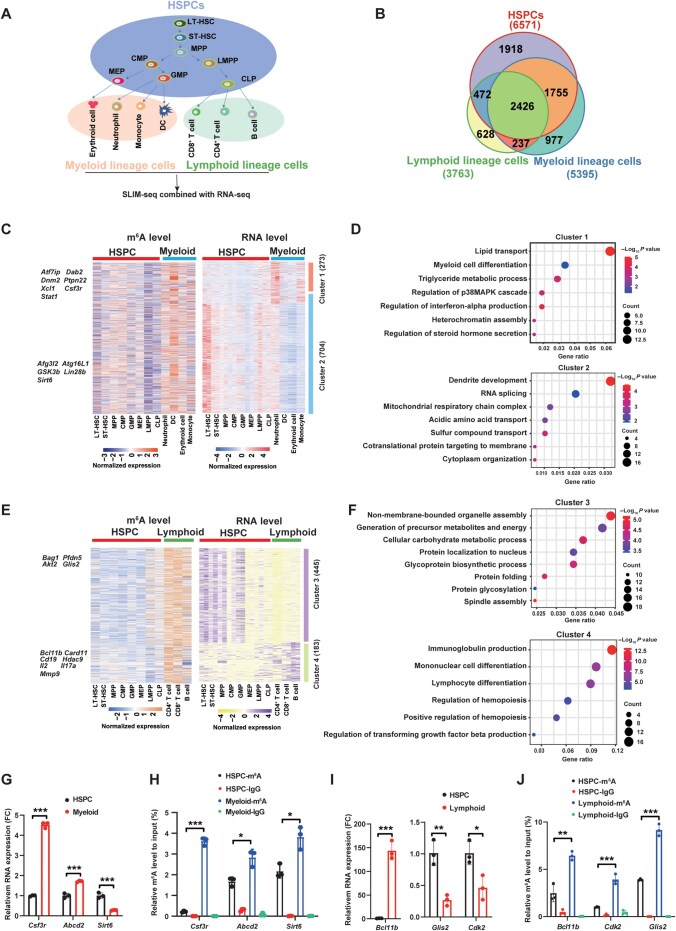
m^6^A modification affects mRNA expression in HSPCs, myeloid lineage cells, and lymphoid lineage cells **A**. Model of the hematopoietic system for m^6^A modification analysis. **B**. Venn diagram showing the overlapping m^6^A targets among HSPCs, myeloid lineage cells, and lymphoid lineage cells. **C**. Heatmap showing the m^6^A (left) and mRNA (right) levels of 977 m^6^A-tagged genes specific in myeloid lineage cells. **D**. Functional enrichment analysis of significantly upregulated (Cluster 1) and downregulated (Cluster 2) differentially expressed genes in myeloid lineage cells relative to their expression levels in HSPCs. **E**. Heatmap showing the m^6^A (left) and mRNA (right) levels of 628 m^6^A-tagged genes specific in lymphoid lineage cells. **F**. Functional enrichment analysis of significantly upregulated (Cluster 4) and downregulated (Cluster 3) differentially expressed genes in lymphoid lineage cells relative to their expression levels in HSPCs. **G**. RT-qPCR analysis showing the mRNA levels of *Csf3r*, *Abcd2*, and *Sirt6* in myeloid lineage cells relative to their expression levels in HSPCs. **H**. MeRIP-PCR analysis of the m^6^A enrichment of mRNAs for *Csf3r*, *Abcd2*, and *Sirt6* in HSPCs. **I**. RT-qPCR analysis showing the mRNA levels of *Bcl11b*, *Glis2*, and *Cdk2* in lymphoid lineage cells relative to their expression levels in HSPCs. **J**. MeRIP-PCR analysis of the m^6^A enrichment of mRNAs for *Bcl11b*, *Glis2*, and *Cdk2* in HSPCs. Data are presented as mean ± SD. *P* values were determined by two-tailed *t*-test and analysis of variance (*, *P* < 0.05; **, *P* < 0.01; ***, *P* < 0.001). mRNA, messenger RNA; HSPC, hematopoietic stem and progenitor cell; SD, standard deviation; RT-qPCR, real-time quantitative PCR; MeRIP-PCR, methylated RNA immunoprecipitation-PCR; MPP, multipotent progenitor cell; GMP, granulocyte–macrophage progenitor; CMP, common myeloid progenitor; MEP, megakaryocyte–erythroid progenitor; LMPP, lymphoid-primed multi-potential progenitor; CLP, common lymphoid progenitor; DC, dendritic cell; RNA-seq, RNA sequencing; FC, fold change.

Considering that the dynamics of m^6^A modification correlate with the cell fate decision and differentiation trajectory of HSPCs [4,44], we explored the potential pathways that might be regulated by m^6^A modification during lineage commitment. We grouped 15 distinct hematopoietic populations into three classes: HSPCs [including LT-HSC, ST-HSC, multipotent progenitor cell (MPP), common myeloid progenitor (CMP), granulocyte–macrophage progenitor (GMP), megakaryocyte–erythroid progenitor (MEP), lymphoid-primed multipotential progenitor (LMPP), and common lymphoid progenitor (CLP)], myeloid lineage cells (including neutrophil, dendritic cell, erythroid cell, and monocyte), and lymphoid lineage cells (CD4^+^ T cell, CD8^+^ T cell, and B cell). These three groups contained 6571, 5395, and 3763 m^6^A-tagged mRNAs, respectively (**Figure 2**A and B). Expectedly, most of the m^6^A targets were shared by two or all of the three groups. Interestingly, we identified 1918 HSPC-specific m^6^A targets, 977 myeloid-specific m^6^A targets, and 628 lymphoid-specific m^6^A targets (Figure 2B), suggesting that these lineage-specific m^6^A targets play an important role in lineage commitment.

Next, we focused on the lineage-specific m^6^A targets and determined their correlation with m^6^A and mRNA levels. A general negative correlation between m^6^A and mRNA levels was observed for most m^6^A targets [72% and 70% for myeloid lineage (Cluster 2) and lymphoid lineage (Cluster 3), respectively]; approximately 28% and 30% of the m^6^A targets for myeloid (Cluster 1) and lymphoid (Cluster 4) lineages, respectively, showed a positive correlation with their mRNA levels (Figure 2C–F). Interestingly, the genes in Cluster 1 exhibited high m^6^A and mRNA levels in myeloid lineage cells, and GO analysis showed that these genes are mainly enriched in the regulation of steroid hormone secretion, heterochromatin assembly, regulation of interferon-α production, myeloid cell differentiation, and lipid transport (Figure 2D). The genes in Cluster 2 showed high m^6^A levels but low mRNA levels in myeloid lineage cells, and they are involved in dendritic development, RNA splicing, mitochondrial respiratory chain complex, and amino acid transport (Figure 2D). Similarly, the genes in Cluster 3 exhibited high m^6^A levels but low mRNA levels in lymphoid lineage cells, and GO analysis showed that these genes are enriched in organelle assembly, metabolic process, and protein glycosylation (Figure 2E and F). By contrast, the genes in Cluster 4 showed both high m^6^A and mRNA levels in lymphoid cells, and they are mainly involved in immunoglobulin production, lymphocyte differentiation, and regulation of transforming growth factor (TGF)-β pathway (Figure 2E and F). Next, we performed real-time quantitative polymerase chain reaction (RT-qPCR) and methylated RNA immunoprecipitation PCR (MeRIP-PCR) analyses and validated several differentially modified m^6^A targets that are key for myeloid (*e.g.*, *Csf3r*, *Sirt6*, and *Abcd2*) or lymphoid (*e.g.*, *Bcl11b*, *Glis2*, and *Cdk2*) lineage differentiation (Figure 2G–J). Together, these results indicate that m^6^A modification is involved in cell fate commitment in myeloid and lymphoid lineages.

### Reprogramming of m^6^A modification occurs in AML development

**Figure 3:**
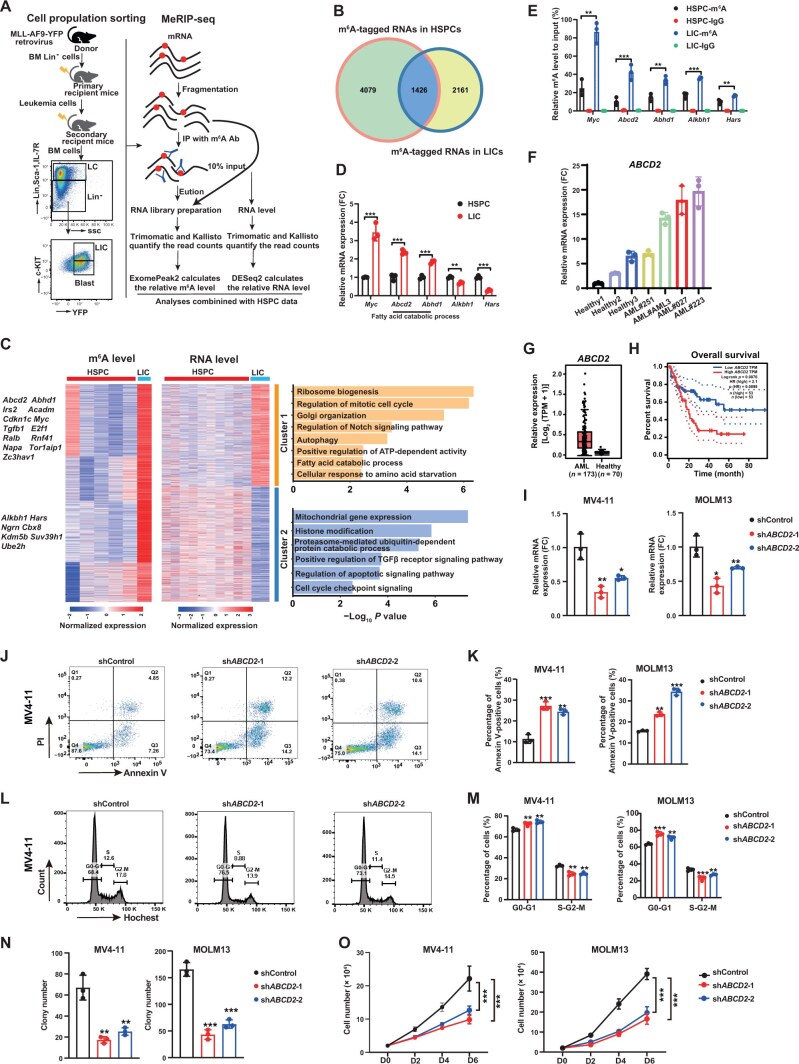
m^6^A modification of *ABCD2* mRNA promotes AML progression **A**. Experimental scheme for generating AML mice model, flow cytometry gating strategy of LICs, and schematic of MeRIP-seq. **B**. Venn diagram showing m^6^A-tagged RNAs shared between HSPCs and LICs. **C**. Comprehensive correlation and GO enrichment analyses of the m^6^A and RNA levels in HSPCs and LICs. Left: heatmap showing the m^6^A and RNA levels of 2161 LIC-specific m^6^A-tagged targets, which were grouped through *K*-means clustering (*K* = 2). Right: GO enrichment analysis results of two clusters. **D**. RT-qPCR analysis showing the mRNA levels of *Myc*, *Abcd2*, *Abhd1*, *Alkbh1*, and *Hars* in AML mice-derived LICs relative to their expression levels in normal HSPCs. **E**. MeRIP-PCR analysis of the m^6^A enrichment of mRNAs for *Myc*, *Abcd2*, *Abhd1*, *Alkbh1*, and *Hars* in AML mice-derived LICs. **F**. RT-qPCR analysis showing *ABCD2* expression in normal BM cells obtained from healthy donors (*n* = 3) and AML patient-derived primary leukemia cells (*n* = 4). **G**. Expression of *ABCD2* mRNA (TPM) in AML patient-derived cells (*n* = 173) and healthy donor blood cells (*n* = 70). **H**. Kaplan-Meier plot of the overall survival of AML patients stratified by *ABCD2* expression level above (*ABCD2*^high^) or below (*ABCD2*^low^) the median value. **I**. RT-qPCR analysis showing *ABCD2* expression in leukemia cells (MV4-11 and MOLM13) after knockdown of *ABCD2* by sh*ABCD2*-1 and sh*ABCD2*-2. **J**. Representative flow cytometry plots showing the apoptotic analysis results for MV4-11 leukemia cells after knockdown of *ABCD2* by sh*ABCD2*-1 and sh*ABCD2*-2. **K**. Apoptotic percentage of leukemia cells after knockdown of *ABCD2* by sh*ABCD2*-1 and sh*ABCD2*-2. **L**. Representative flow cytometry plots showing the cell cycle analysis of MV4-11 leukemia cells after knockdown of *ABCD2* by sh*ABCD2*-1 and sh*ABCD2*-2. **M**. Cell cycle analysis showing the percentages of leukemia cells in different cell cycle phases after knockdown of *ABCD2* by sh*ABCD2*-1 and sh*ABCD2*-2. **N**. Colony-forming unit assay showing the clonogenic defects of leukemia cells (MV4-11 and MOLM13) after knockdown of *ABCD2* by sh*ABCD2*-1 and sh*ABCD2*-2. **O**. Growth curves of MOLM13 and MV4-11 leukemia cells after knockdown of *ABCD2* by sh*ABCD2*-1 and sh*ABCD2*-2. Data are presented as mean ± SD. *P* values were determined by two-tailed *t*-test or one-way analysis of variance (*, *P* < 0.05; **, *P* < 0.01; ***, *P* < 0.001). LC, leukemia cell; LIC, leukemia-initiating cell; AML, acute myeloid leukemia; SSC, side scatter; ABCD2, ATP-binding cassette subfamily D member 2; MeRIP-seq, methylated RNA immunoprecipitation sequencing; TPM, transcripts per kilobase per million mapped reads; HR, hazard ratio; PI, propidium iodide; D, day.

Considering that m^6^A is a key player in AML development and LIC maintenance [28,33,35,36,45–48], we investigated the dynamics of m^6^A methylome during leukemogenesis. Recently, we established the m^6^A methylome of LICs from MLL-AF9-induced AML [43]. Using this m^6^A methylome of LICs, we further analyzed the difference in the m^6^A methylomes between LICs and normal HSPCs (**Figure 3**A). Intriguingly, we found that 39.75% of the m^6^A targets (1426 out of 3587) in LICs were also found in HSPCs and that approximately 60.25% (2161 out of 3587) were LIC-specific (Figure 3B), suggesting that m^6^A modification facilitates reprogramming that promotes cellular transformation during leukemogenesis.

Next, we focused on the 2161 m^6^A targets with obviously increased m^6^A levels in LICs and attempted to identify the biological processes affected by m^6^A modification during leukemogenesis. Expectedly, after taking into account the changes in mRNA levels, we observed two patterns: positive correlation in Cluster 1 and negative correlation in Cluster 2. The genes in Cluster 1 displayed high m^6^A and mRNA levels in LICs, and GO analysis showed that these genes are mainly enriched in ribosome biogenesis, mitotic cell cycle regulation, Golgi organization, autophagy, fatty acid metabolism, and cellular response to amino acid starvation (Figure 3C). The fatty acid catabolic process-related genes (*e.g.*, *Abcd2*, *Abhd1*, *Acadm*, and *Irs2*) showed higher m^6^A and mRNA levels in LICs than in normal HSPCs, indicating that fatty acid metabolism is key in promoting LIC transformation. By contrast, the genes in Cluster 2 showed high m^6^A levels but decreased mRNA expression in LICs, and they are involved in mitochondrial gene expression, histone modification, proteasome-mediated protein catabolic process, regulation of apoptosis pathway, cell cycle checkpoint signaling, immunoglobulin production, lymphocyte differentiation, and regulation of TGF-β pathway (Figure 3C). m^6^A-RIP-PCR and RT-qPCR analyses validated these differentially modified m^6^A targets (Figure 3D and E). Our data collectively indicate that reprogramming of RNA m^6^A modification occurs during AML development.

### ATP-binding cassette subfamily D member 2, a very long chain fatty acid transporter, promotes leukemogenesis

AML exhibits an altered metabolic adaptation characterized by increased mitochondrial mass and a great reliance on oxidative phosphorylation and fatty acid oxidation, which distinguish leukemic cells and LICs from normal HSCs [49]. A recent study indicated that very long chain fatty acid (VLCFA) metabolism is required in AML [50]. Interestingly, among the fatty acid catabolic process-related genes mentioned above, ATP-binding cassette subfamily D member 2 (ABCD2, also called ALDRP) is the peroxisomal VLCFA transporter and is involved in lipid metabolism via peroxisomal beta-oxidation [51]. However, the role of ABCD2 in AML remains unknown. We first assessed *ABCD2* expression in AML patients, and we found significantly higher *ABCD2* expression levels in AML patients than in healthy controls (Figure 3F and G). Importantly, the elevated *ABCD2* expression correlated with poor survival of AML patients (Figure 3H). Next, we knocked down *ABCD2* in two human leukemia cell lines, MV4-11 and MOLM13 (Figure 3I). We found that *ABCD2* knockdown significantly inhibited the proliferation of human AML cell lines (MOLM13 and MV4-11) and induced their apoptosis (Figure 3J–M). Moreover, *ABCD2* knockdown obviously inhibited the colony-forming and cellular growth abilities of these two cell lines (Figure 3N and O). Together, these results indicate that ABCD2 is required in AML development.

### m^6^A readers recognize different m^6^A targets in LICs

**Figure 4:**
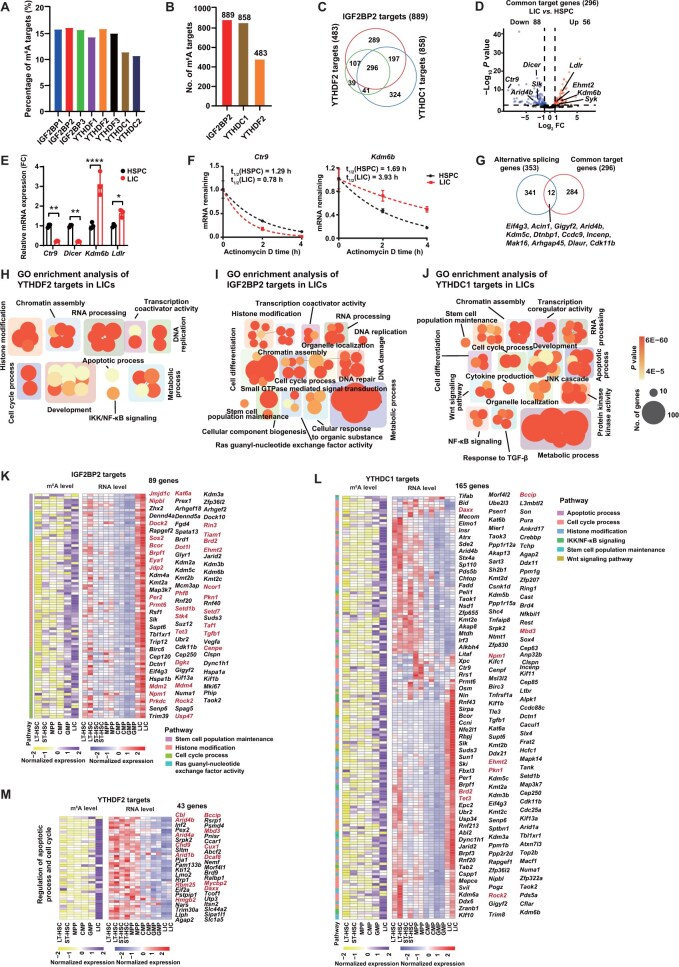
m^6^A readers recognize different m^6^A targets in LICs and participate in multiple biological processes **A**. Bar plot showing the percentage of targets recognized by m^6^A readers (IGF2BP1/2/3, YTHDF1/2, and YTHDC1/2) in the m6^A^ methylome of LICs. The targets were defined as the overlapping mRNAs between the high-confidence m^6^A-tagged mRNAs identified in LICs and the known targets of m^6^A readers (IGF2BP1/2/3, YTHDF1/2, and YTHDC1/2) in human cell lines. **B**. Bar plot showing the number of targets recognized by m^6^A readers (IGF2BP2, YTHDC1, and YTHDF2) in the m^6^A methylome of LICs. The targets were defined as the overlapping mRNAs between the high-confidence m^6^A-tagged mRNAs identified in LICs and the known targets of m^6^A readers (IGF2BP2, YTHDC1, and YTHDF2) in primary murine LICs. **C**. Venn diagram showing the common targets of m^6^A readers (IGF2BP2, YTHDF2, and YTHDC1) in LICs. **D**. Volcano plots showing the differentially expressed genes in the 296 common targets (LICs *vs.* HSPCs). **E**. RT-qPCR analysis showing the relative mRNA levels of *Ctr9*, *Dicer*, *Kdm6b*, and *Ldlr*. **F**. mRNA half-life (t_1/2_) of *Ctr9* and *Kdm6b* in HSPCs and LICs. **G**. Venn diagram showing the differential splicing events during a specific HSC transition (FDR < 0.05 and IncLevelDifference > 0.1). **H**.–**J**. GO enrichment analysis of the targets of m^6^A readers YTHDF2 (H), IGF2BP2 (I), and YTHDC1 (J) in LICs. **K**.–**M**. Heatmaps showing the m^6^A and RNA levels of the 89, 165, and 43 targets of IGF2BP2 (K), YTHDC1 (L), and YTHDF2 (M) in HSPCs and LICs, respectively. FDR, false discovery rate.

Considering that m^6^A sites are recognized by different m^6^A readers [17,52], we explored how m^6^A-modified mRNAs are regulated by m^6^A readers in LICs. First, we integrated publicly available RNA-immunoprecipitation sequencing (RIP-seq) or individual-nucleotide resolution ultraviolet crosslinking and immunoprecipitation high-throughput sequencing (iCLIP-seq) datasets to analyze the distribution of the targets of IGF2BP1/2/3, YTHDF1/2/3, and YTHDC1/2 in the m^6^A methylome of LICs. We found that similar percentages of m^6^A targets in LICs are recognized by different m^6^A readers, except those for YTHDC1/2 (**Figure 4**A). Given that the datasets were used to identify the targets of m^6^A readers in HEK293T cells, to be more accurate, we chose one representative member from each of these m^6^A reader families (IGF2BP2, YTHDF2, and YTHDC1) and performed an RIP-seq analysis using primary murine LICs. We identified 889, 858, and 483 m^6^A targets in LICs for IGF2BP2, YTHDC1, and YTHDF2, respectively (Figure 4B). Interestingly, we found that these three readers recognized a total of 1293 targets, which constitute approximately 36% of the m^6^A-tagged mRNAs identified in LICs (1293 out of 3587). Moreover, approximately 22% of the targets (296 out of 1293) were shared by these three readers, and approximately 50% were reader-specific (652 out of 1293) (Figure 4C). We further analyzed the changes in the 296 common targets in terms of mRNA stability, splicing, and degradation during the transformation of HSCs into LICs. Of the 296 targets, 56 displayed higher expression levels in LICs than in normal HSPCs (Figure 4D), indicating that m^6^A modification increases the stability of these mRNAs. By contrast, 88 of these 296 targets showed lower expression levels in LICs than in HSPCs (Figure 4D), suggesting that m^6^A modification facilitates the degradation of these mRNAs. These findings are in line with previous results [53–56] wherein tumor suppressor genes, such as *Ctr9* and *Dicer*, are lowly expressed in LICs, whereas *Kdm6b* and *Ldlr*, which play oncogenic roles, are highly expressed in LICs (Figure 4E). An RNA decay assay confirmed the change in *Ctr9* and *Kdm6b* mRNA stability in LICs and HSPCs (Figure 4F). These results suggest that the fates of these common m^6^A mRNAs are closely associated with their biological functions, although they are recognized by different readers. Moreover, we observed that 12 out of these 296 targets showed differential splicing during transformation of HSCs into LICs (Figure 4G). Together, these results suggest that different m^6^A readers recognize the same m^6^A targets; at the same time, they exert their unique function by reading specific m^6^A targets.

To further explore the roles of m^6^A readers in regulating LIC function, we compared the biological processes and functions of the m^6^A targets. Intriguingly, some common biological processes, such as histone modification, chromatin assembly, RNA processing, transcription coactivator activity, and cell cycle regulation, were shared and enriched by the m^6^A targets of all three readers (IGF2BP2, YTHDF2, and YTHDC1) (Figure 4H–J). However, the targets of IGF2BP2 and YTHDF2 are also enriched in DNA replication pathways, whereas those of IGF2BP2 and YTHDC1 are mainly involved in cell differentiation, organelle localization, and stem cell population maintenance. Interestingly, YTHDF2’s targets preferentially participate in histone modification and RNA processing, IGF2BP2’s targets are mainly enriched in metabolic process and cell differentiation, and YTHDC1’s targets are mainly involved in metabolic process. Figure 4K shows the 89 IGF2BP2 target genes (*e.g.*, *Jmjd1c*, *Kat6a*, *Nipbl*, *Prex1*, and *Prmt6*) which were highly expressed with high m^6^A levels in LICs. For YTHDC1, 165 target genes (*e.g.*, *Tifab*, *Morf4l2*, *Bccip*, *Bid*, *Daxx*, and *Tet3*) exhibited either positive or negative correlation between their m^6^A and mRNA levels in LICs (Figure 4L). For YTHDF2, 43 targets (*e.g.*, *Cbl*, *Arid4b*, *Pex2*, *Chd9*, and *Lmo2*) displayed low expression with high m^6^A levels in LICs (Figure 4M). Many of these target genes have been shown to regulate leukemia development. For instance, a recent study demonstrated that the histone acetyltransferase KAT6A forms an epigenetic transcriptional control module with acetyl-lysine reader ENL and drives critical leukemogenic gene expression program, such as myeloid differentiation [57]. Our recent work indicated that the protein arginine methyltransferase PRMT6 maintains LIC function by catalyzing H3R2me2a and suppressing the expression of the lipid transporter MFSD2A [43]. JMJD1C, a jumonji C-containing H3K9 demethylase, is a critical regulator of aberrant metabolic processes in homeobox A9-dependent AML [58]. Overall, these findings suggest that different m^6^A readers play important roles in leukemia by participating in various biological processes.

## Discussion

As accumulating evidence demonstrates that RNA m^6^A plays much more complex roles in physiological and pathological contexts, it becomes necessary to perform m^6^A profiling and explore the role of m^6^A in hematopoiesis and leukemogenesis. Through a comparative analysis of the RNA m^6^A methylome of normal HSPCs and LICs, this study reveals the occurrence of reprogramming of RNA m^6^A modification in leukemogenesis and identifies ABCD2 as a key factor for AML development.

Our findings provide insights into how RNA m^6^A modification regulates the transcriptional state and lineage commitment of HSCs. For instance, we found that m^6^A regulates the expression of Notch pathway-related genes in LT-HSCs. This finding is in line with that of a previous work showing that m^6^A regulates Notch signaling to determine cell fate and guides the earliest HSPCs during endothelial-to-hematopoietic transition (EHT) in zebrafish embryogenesis [59]. In addition, this work focuses on the abrupt change in m^6^A modification during the transition of LT-HSCs into ST-HSCs, and it reveals that during this transition, several biological processes, including vitamin metabolism and histone modification, are regulated by m^6^A. These are novel findings. Although a recent study showed that vitamin A–retinoic acid signaling regulates HSC dormancy [60], how this vitamin metabolism is regulated in HSCs remains unclear. Our work suggests that RNA m^6^A plays an important role in regulating vitamin metabolism in HSCs. This finding is quite interesting, and it extends a previous observation and provides new insights into the transition of LT-HSCs into ST-HSCs. Moreover, further comparison of the m^6^A profiles between two consecutive differentiation stages would provide more information about m^6^A modification in relation to the regulation of cellular fates.

Our results suggest that in leukemogenesis, reprogramming of RNA m^6^A modification occurs during cellular transformation. We found that most m^6^A modifications are newly established and are LIC-specific. Moreover, these m^6^A-tagged targets are involved in several biological processes during leukemogenesis. For instance, fatty acid catabolic process-related genes exhibit higher m^6^A and mRNA levels in LICs than in normal HSPCs, suggesting that fatty acid metabolism plays a key role in LIC transformation. Fatty acid metabolism underlies chemotherapy resistance in AML stem cells [61]. However, the mechanism of how RNA m^6^A regulates fatty acid metabolism in LICs warrants further investigation. Furthermore, our RIP-seq data for LICs reveal that different readers may share the same m^6^A targets or may recognize different m^6^A targets. This flexibility makes RNA m^6^A modifications more complex. Thus, clarifying the role of each m^6^A reader in AML development is necessary. In addition, it seems that only a few m^6^A-tagged mRNAs exert their functions under certain physiological and pathological conditions. How m^6^A specificity is determined remains a question. Moreover, how transcripts are selected by m^6^A writers, erasers, and readers remains unclear. Diverse regulatory machineries can be recruited to m^6^A-tagged mRNAs through m^6^A readers, and RNA-binding proteins involved in these machineries might lead to the specificity of m^6^A readers toward certain m^6^A sites or m^6^A-tagged RNAs. Therefore, identifying the cofactors of m^6^A modifiers may help us to answer the aforementioned questions in the future. Collectively, our results reveal a critical role of m^6^A modification in normal hematopoiesis and leukemogenesis and provide a comprehensive understanding of m^6^A function in regulating cell state transitions.

## Materials and methods

### Primary AML patient and cord blood samples

AML patient samples were collected from bone marrow (BM) aspirations with informed consent. Mononuclear cells (MNCs) were isolated by density gradient centrifugation with Ficoll (Catalog No. 17-1140-02, GE Healthcare Life Science, Marlborough, MA).

### Cell lines

The human acute leukemia cell lines (MOLM13 and MV4-11) were cultured in Roswell Park Memorial Institute 1640 Medium (RPMI-1640; Catalog No. SH30022.01, HyClone, Logan, UT) or Iscove’s Modified Dulbecco’s Medium (IMDM; Catalog No. SH30228.01, HyClone) with 10% fetal bovine serum (FBS; Catalog No. 10270-106, Gibco, Grand Island, NY) and 1% penicillin–streptomycin (Catalog No. P4333, Sigma-Aldrich, St. Louis, MO). HEK293T cells were cultured in Dulbecco’s modified eagle medium (DMEM; Catalog No. SH30022.01, HyClone), supplemented with 10% FBS and 1% penicillin–streptomycin. Target cells were infected with the virus in the presence of 8 mg/ml polybrene (Catalog No.28728-55-4, Sigma-Aldrich) under centrifugation at 2200 r/min. Media were changed at 12 h after infection, and 2 mg/ml puromycin (Catalog No. 58-58-2, Sigma-Aldrich) was added at 48 h after infection if selection was needed.

### Cell proliferation and colony-forming unit assays

Proliferation assays of MOLM13 and MV4-11 were performed as follows: cells were transduced with indicated lentivirus. After 48-h selection with 2 mg/ml puromycin, 20,000 cells were plated into 12-well plates in triplicates. Cells were counted every two days. For colony-forming assays, 1000 transduced cells were plated in 1.2% methylcellulose medium (supplemented with 1% penicillin–streptomycin and 10% FBS).

### Methylated RNA immunoprecipitation sequencing data preprocessing and analysis

The methylated RNA immunoprecipitation sequencing (MeRIP-seq) dataset (GEO: GSE210280) was generated in our previous work [42,43]. The process of analysis was performed as previously described. In brief, the reads from mRNA input and MeRIP-seq libraries were aligned to mm10 mouse reference genome with HISAT2 (v2.2.1). The m^6^A peaks were called by exomePeak2 (v1.0.0). The distribution of m^6^A peaks over different regions on the transcript was depicted with annotations generated by Guitar (v2.4.0). RNA sequence motifs enriched in m^6^A peaks were identified with HOMER (v4.11). For calculating the relative m^6^A level for each gene, we used the mean of log_2_ fold change (FC) for all peaks annotated to genes, and then we identified high-confidence m^6^A-tagged genes by setting up the cutoff line based on log_2_ FC [immunoprecipitation (IP) *vs.* mRNA input] > 1 and *P* < 0.001. We further calculated the Z-score of adjusted FC in each cell type, and this Z-score represents the relative m^6^A level. The reads from the raw sequencing data of mRNA input samples were preprocessed using Trim Galore, and Kallisto was used to quantify the read counts for each transcript (GRCm38, complementary DNA sequences). The read counts of mRNA input were used as the mRNA level.

### SLIM-seq data preprocessing and analysis

The SLIM-seq dataset (GEO: GSE165863) was generated in our previous work [43]. The raw sequencing data of all input and IP samples were preprocessed using Trimmomatic, and Kallisto was used to quantify the read counts for each transcript. During analysis, we performed fit analysis using the negative binomial distribution for each sample, and further ran generalized linear model (GLM) to eliminate the deviation caused by the transcriptional difference. The read counts for genes were assessed by R package tximport. For calculating the relative m^6^A level for each gene, we used DESeq2 to compare the read counts of genes between IP samples and input samples on a transcriptome-wide scale. Based on the adjusted FC (Wald statistic, fourth column of output of DESeq2) of each gene from the output of DESeq2, we further calculated the Z-score of adjusted FC in each cell type, and this Z-score represents the relative m^6^A level. We identified high-confidence m^6^A-tagged mRNAs by setting up the cutoff line based on read count > 1 in input samples, log_2_ FC (IP *vs.* input) > 0, and *P* < 0.05. If the *P* value equals ‘‘NA’’ in output of DESeq2, we identified high-confidence m^6^A-tagged genes with log_2_ FC > 2.

### RNA sequencing data preprocessing and analysis

The RNA sequencing (RNA-seq) datasets (GEO: GSE165863 and GSE210280) were generated in our previous work [42,43]. All raw sequencing data were preprocessed using fastp, and Kallisto was used to quantify the read counts for each transcript (GRCm38, complementary DNA sequences). The read counts for genes were assessed by R package tximport. We used DESeq2 to analyze differential genes between wild-type and knockout samples, and the differentially expressed genes were identified with *P* < 0.05 and |log_2_ FC| > 0.5.

### RIP-seq data processing and analysis

The RIP-seq dataset (GEO: GSE218610) was generated in our previous work [43]. All raw sequencing data were preprocessed using Trim Galore, and then mapped to the mouse genome reference (mm10) using HISAT2 (v2.2.1). Peaks were called with MACS2 (v2.2.7.1). Then we identified high-confidence peaks by setting up the cutoff line based on FC > 4 and *P* < 0.01. The peaks were annotated to genes by clusterProfiler (v3.16.0).

### RT-qPCR

Total RNA was isolated using TRIzol reagent (Catalog No. 9109, Takara, Shiga, Japan). Then, 1 µg of purified total RNA was retrotranscribed using the ReverTra Ace qPCR RT Kit (Catalog No. FSQ-201, TOYOBO, Shanghai, China). The levels of specific RNAs were measured using Bio-Rad Real-Time PCR Systems and Fast SYBR Green PCR Master Mix (Catalog No. 1725120, Bio-Rad, Hercules, CA) according to the manufacturer’s instructions. Primer sequences are listed in Table S1. The 2^−ΔΔCt^ method was used to normalize expression to glyceraldehyde-3-phosphate dehydrogenase (*GAPDH*) and beta-actin (*ACTB*) for cell lines.

### Super-low-input m^6^A quantitative PCR

m^6^A MeRIP is based on the SLIM-seq protocol described in a previous study [4]. All primer sequences are listed in Table S1. mRNA quantification from the MeRIP samples was carried out using the 2^−ΔΔCt^ method against non-immunoprecipitated input RNA controls.

### *ABCD2* knockdown assay

For *ABCD2* knockdown, the short hairpin RNA (shRNA) sequences were cloned into the pLKO.1 vector according to the manufacturer’s instructions. All the shRNA sequences are listed in Table S2. Lentiviruses were produced in HEK293T cells cotransfected with the shRNA constructs and viral packaging constructs (pMD2.G and psPAX2) using polyethylenimine. Viral supernatants were harvested at 36 h and 50 h after transfection.

### Flow cytometry and cell sorting

Mouse BM cells were isolated from tibia and femur, and resuspended in red blood cell lysis buffer for 3 min on ice to remove red blood cells, and washed with phosphate buffer saline (PBS) (containing 2% FBS). Total BM cells were stained with B220 for B cells, CD4 for CD4^+^ T cells, CD8 for CD8^+^ T cells, Gr-1 for granulocytes, Mac-1 for monocyte cells, Ter119 for erythrocyte cells, and CD11c for DCs. For HSPC isolation, BM cells were incubated with biotin-labeled lineage antibody cocktail and Lin^−^ immature cells were first enriched using the EasySep Mouse Hematopoietic Progenitor Cell Isolation Kit (Catalog No. 19856, STEMCELL, Vancouver, Canada) according to the manufacturer’s instructions. After washing, cells were stained with the fluorochrome-conjugated secondary antibody Streptavidin APC-eFluor 780 (Catalog No. 405208, BioLegend, San Diego, CA) for biotin detection and PE-c-Kit (Catalog No. 12-1172-83, eBioscience, San Diego, CA).

### RNA decay assay

LICs and HSPCs were treated with actinomycin D (Catalog No. A9415, Sigma-Aldrich) at a final concentration of 5 mg/ml for indicated time and then collected. Total RNA at 0, 2, and 4 h was extracted using TRIzol reagent (Catalog No. 9109, Takara), and the relative expression was detected by RT-qPCR.

### Enrichment analysis

We performed GO enrichment analysis for m^6^A-modified genes using clusterProfiler (v3.16.0). For better visualization, Cytoscape (v3.9.1) was used to generate manually defined layout and export the final graph.

## Ethical statement

All experiments referring to human samples were conducted in compliance with all relevant ethical regulations, and were approved by the Medical Ethics Committees of School of Medicine, Wuhan University, China (Approval No. WAFF-2022-0066).

## CRediT author statement

**Weidong Liu:** Software, Validation, Formal analysis, Resources, Investigation, Data curation, Writing – original draft, Writing – review & editing. **Yuhua Wang:** Validation, Data curation, Writing – original draft, Writing – review & editing. **Shuxin Yao:** Resources, Investigation. **Guoqiang Han:** Software, Investigation, Data curation. **Jin Hu:** Resources, Investigation. **Rong Yin:** Methodology, Resources. **Fuling Zhou:** Resources. **Ying Cheng:** Software, Validation, Formal analysis, Investigation, Writing – original draft. **Haojian Zhang**: Writing – review & editing, Supervision, Funding acquisition. All authors have read and approved the final manuscript.

## Competing interests

The authors have declared no competing interests.

## Supplementary Material

qzae049_Supplementary_Data
