# Supplementary material for: Reprogramming of RNA m6A Modification Is Required for Acute Myeloid Leukemia Development
Source: Genomics Proteomics Bioinformatics. 2024 Jun 24;23(2):qzae049. doi: 10.1093/gpbjnl/qzae049 (PMC12373641; doi:10.1093/gpbjnl/qzae049)
Supplement: qzae049_Supplementary_Data [file qzae049_supplementary_data.zip › supplementary material captions.docx]

**Supplementary materials**

**Table S1 Primer sequences for qRT-PCR and SLIM-qPCR**

**Table S2 Primer sequences for shRNAs**
